# Genome Sequence and Host Range Investigation of Cluster CV Phage Spink

**DOI:** 10.17912/micropub.biology.002116

**Published:** 2026-06-01

**Authors:** Ethan Smith, Ailanys E. Cordero-Morales, Elizabeth Padilla-Crespo, Ek Han Tan, Nathan Reyna, Ruth Plymale

**Affiliations:** 1 Ouachita Baptist University, Arkadelphia, AR, US; 2 Inter American University of Puerto Rico, Aguadilla Campus, Aguadilla, PR, US; 3 School of Biology and Ecology, University of Maine, Orono, ME, US; 4 Howard Payne University, Brownwood, TX, US

## Abstract

Bacteriophage Spink was isolated using host bacterium *Gordonia terrae* 3612. Spink's host range was investigated across six other bacterial strains; however, its host range appeared restricted to *G. terrae* , as all expansion host pairings resulted in killing from without (KFW) or no infection (NI). Spink displays a siphovirus morphotype and possesses a 49,479 bp genome. Based on gene content similarity, Spink is assigned to cluster CV. Putative functions were assigned to 39 of 78 genes. Like most CV phages, Spink is temperate and encodes several host interaction genes, including a tyrosine integrase, immunity repressor, and a RelE-RelB toxin-antitoxin pair.

**Figure 1. Spink Isolation, Visualization, and Host Range Investigation Data:** **Figure 1 –** (A) Spink Virion Morphology – Transmission electron micrograph (TEM) of *Gordonia* phage Spink. Phage lysate was negatively stained with 1% uranyl acetate and viewed using a Tecnai F20 TEM, with image taken at 80 kV and 80,000× magnification on a Gatan Eagle camera. Scale bar: 100 nm. (B) Turbid plaques formed by bacteriophage Spink on a lawn of *Gordonia terrae* 3612 during its initial isolation from soil collected in Arkadelphia, Arkansas in 2017. (C) Representative turbid plaque formed by bacteriophage Spink on *Gordonia* strain CAG3 (Isolation Host), demonstrating productive infection. (D) Plate showing killing from without (KFW) activity of bacteriophage Spink when applied to the RUBY host strain. Clearing of the bacterial lawn is observed without the formation of discrete plaques, indicating high-titer phage-mediated lysis rather than productive infection. **Table 1 –** Average plaque-forming units per milliliter (pfu/mL) of Spink on different *Gordonia* host strains across three rounds of isolation. NI = no infection observed; KFW = killing from without.

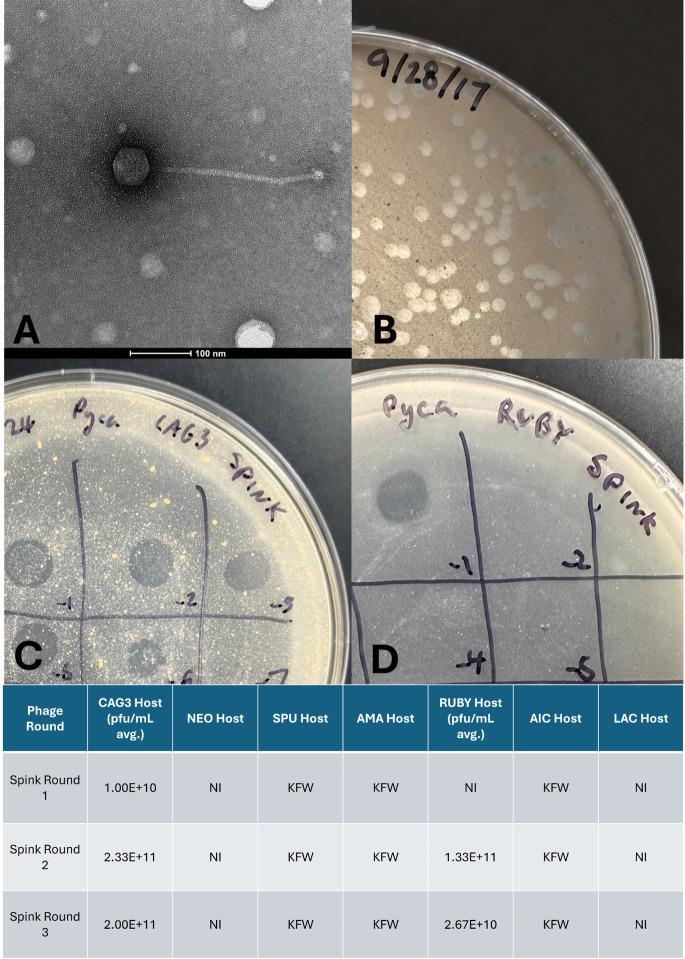



## Description

Bacteriophage Spink was isolated in 2017 from soil in Arkadelphia, Arkansas (GPS: 34.1258° N, 93.0532° W), using standard protocols for enriched isolation as described (Poxleitner et al., n.d.). Soil samples were washed in peptone-yeast extract-calcium (PYCa) liquid medium for two hours at 30°C with shaking to suspend phage particles. The suspension was then filtered through a 0.22 µm filter. The filtrate was then inoculated with *Gordonia terrae* and incubated at 30°C with shaking for three or four days before being filtered and plated. Phage Spink went through three rounds of plaque purification. Phage replication produced turbid plaques with a diameter of 1 mm. Viewed by negative-stain TEM, phage Spink showed a siphovirus morphotype. Capsid diameter and tail length were measured with ImageJ v1.53k (Schneider et al., 2012) and revealed a capsid diameter of 60.2 ± 0.8 nm and tail length of 285 ± 3 nm.

The host range of phage Spink was investigated by performing spot titers of the phage on the following Gordonia species: *G. rubripertincta* (NRRL B-16540 SEA), *Gordonia lacunae* (NRRL B-24551 SEA), *Gordonia aichiensis* (NRRL B-16934), *Gordonia amarae* (NRRL B-18176), *Gordonia neofelifaecis* (NRRL B-59395), and *Gordonia sputi* (NRRL B-16936). Spink showed a productive infection only on *G. terrae* (Fig. 1).

Genomic DNA was extracted from the phage lysate using the Promega Wizard DNA cleanup kit. Sequencing of Spink DNA was performed on an Oxford Nanopore Technologies MinION platform using a FLO-MIN106 flow cell. The library was prepared using the SQK-LSK109 ligation sequencing kit according to the manufacturer's specifications. Sequencing produced 2,750 reads and 16.39 Mb of data over five hours. Basecalling of the raw FAST5 files was performed using Guppy v6.5.7 (Wick et al., 2019) with default parameters for FLO-MIN106 and SQK-LSK109. The reads were then filtered using Filtlong v2.0 (Wick, 2017) with the following parameters: mean quality weight of 30, minimum read length of 2,000 bp, and target bases of 5,000,000. This resulted in 1,537 filtered reads totaling 13,221,164 bases.

The filtered reads were then assembled to a genome length of 49,479 bp (~267× coverage) using Flye v2.8.3 (Kolmogorov et al., 2019) with the following parameters: --nano-raw, 100 kb estimated genome size, 1,000 bp minimum overlap, four threads, and haplotype retention enabled. The assembly was followed by polishing via Medaka v2.0.1 (Oxford Nanopore Technologies, 2022). The genome's GC content of 67.4% is similar to its isolation host *Gordonia terrae* 3612 (67.8%) (Russell et al., 2016).

The Spink genome was auto-annotated using Glimmer v3.02 (Delcher et al., 2007) and GeneMark v2.5 (Besemer et al., 2005). It was then refined through manual annotation with DNA Master v5.23.6 (Lawrence, 2007), PECAAN v20241104 (Rinehart et al., 2024), BLAST (Camacho et al., 2009), DeepTMHMM v1.0.24 (Hallgren et al., 2022), and HHpred (Söding et al., 2005). Databases accessed by these programs include the following: BLAST used the Actinobacteriophage database (Russell and Hatfull, 2017) and the NCBI non-redundant database; HHpred used PDB_mmCIF70 and Pfam v36; and the NCBI Conserved Domain Database v3.20. Aragorn v1.2.41 (Laslett, 2004) detected no tRNA genes. All software was used with default parameters.

Throughout annotation, functions were assigned to 39 of 78 annotated genes in the 49,479 bp genome. The majority of genes are transcribed rightward, with ten leftward-transcribed genes located from genes 33 to 44. As with all cluster CV phages, Spink encodes an immunity repressor and a tyrosine integrase, suggesting Spink is able to establish lysogeny. Spink also encodes a RelE-like toxin and RelB-like antitoxin adjacent to its tyrosine integrase gene. Spink was found to encode six minor tail proteins along with three membrane proteins of unknown function containing two or more membrane regions.

**Nucleotide Sequence Accession Numbers**

Sequence data of phage Spink are available under SRA accession SRX28895214 , BioProject PRJNA1240829 , and BioSample SAMN48363775 .
